# S02-4 Physical activity and screen time during the COVID-19 lockdown in Europe: repeated cross-sectional study in 10 countries

**DOI:** 10.1093/eurpub/ckac093.009

**Published:** 2022-08-29

**Authors:** Viktoria A Kovacs, Mirko Brandes, Thomas Suesse, Rok Blagus, Tamas Csanyi, Monika Kaj, Gregor Starc, Paulo Rocha, Anthony D Okely

**Affiliations:** 1 Hungarian School Sport Federation, Budapest, Hungary; 2 Leibniz Institute for Prevention Research and Epidemiology (BIPS), Bremen, Germany; 3 School of Mathematics and Applied Statistics, University of Wollongong, Australia; 4 Institute for Biostatistics and Medical Informatics, Medical Faculty, University of Ljubljana; 5 University of Ljubljana, Ljubljana, Slovenia; 6 Portuguese Institute of Sport and Youth, Lisbon, Portugal; 7 Early Start and Illawarra Health & Medical Research Institute, University of Wollongong, Australia

**Keywords:** Physical activity, surveillance, adolescents, policy

## Abstract

**Background:**

To date, few multi-country data on how the COVID-19 pandemic and restrictions affected children’s physical activity have been published, and most reports originate outside Europe. This repeated cross-sectional study investigated the prevalence and correlates of physical activity (PA) and screen time (ST) in a large sample of European children during the COVID-19 pandemic in January-February 2021, and compared the data to the first lockdown in May-June 2020.

**Methods:**

Data from two online survey rounds were analysed. A total of 8,395 children aged 6-18 years were included in Round 1 (May-June 2020) and 24,302 in Round 2 (Jan-Feb 2021). PA and ST were assessed by 7-day recall measure.

**Results:**

Overall, 9.3% of children (95%CI, 6.9-11.7) met the WHO PA recommendation, which is half of what was observed in Spring 2020 (19.0% [18.2-19.9]). Exceeding ST recommendations was also prevalent in both data collection rounds. Playing outdoors more than 2 hours/day, following a daily routine and being active in online P.E. increased the odds of healthy levels of physical activity and screen time. We also observed a large variability in curriculum time allocated for P.E. In many countries this was lower than the compulsory requirements.

**Conclusions:**

Findings suggest that lockdown in winter has greater negative impact than in spring. Promoting safe and responsible outdoor activities, safeguarding P.E. lessons during distance learning and setting pre-planned, consistent daily routines are important in helping children maintain healthy active lifestyle in pandemic situation. These factors should be prioritised by policymakers, schools and parents.

